# Search for Global Minimum Structures of ${\text{P}}_{2n+1}^{+}$ (*n* = 1–15) Using xTB-Based Basin-Hopping Algorithm

**DOI:** 10.3389/fchem.2021.694156

**Published:** 2021-07-26

**Authors:** Min Zhou, Yicheng Xu, Yongliang Cui, Xianyi Zhang, Xianglei Kong

**Affiliations:** ^1^ School of Physics and Electronic Information, Anhui Normal University, Wuhu, China; ^2^ The State Key Laboratory and Institute of Elemento-Organic Chemistry, Collage of Chemistry, Nankai University, Tianjin, China; ^3^ Collaborative Innovation Center of Chemical Science and Engineering, Nankai University, Tianjin, China

**Keywords:** global optimization, atomic clusters, basin-hopping algorithm, phosphorus cluster cations, xTB method, pnicogen bond

## Abstract

A new program for searching global minimum structures of atomic clusters using basin-hopping algorithm based on the xTB method was developed here. The program can be performed with a much higher speed than its replacement directly based on DFT methods. Considering the structural varieties and complexities in finding their global minimum structures, phosphorus cluster cations were studied by the program. The global minimum structures of cationic ${\text{P}}_{2n+1}^{+}$ (*n* = 1–15) clusters are determined through the unbiased structure searching method. In the last step, further DFT optimization was performed for the selected isomers. For ${\text{P}}_{2n+1}^{+}$ (*n* = 1–4, 7), the found global minimum structures are in consistent with the ones previously reported; while for ${\text{P}}_{2n+1}^{+}$ (*n* = 5, 6, 8–12), newly found isomers are more energy-favorable than those previously reported. And those for ${\text{P}}_{2n+1}^{+}$ (*n* = 13–15) are reported here for the first time. Among them, the most stable isomers of ${\text{P}}_{2n+1}^{+}$ (*n* = 4–6, 9) are characterized by their C_3v_, C_s_, C_2v_ and C_s_ symmetry, in turn. But those of ${\text{P}}_{2n+1}^{+}$ (*n* = 7, 8, 10–12), no symmetry has been identified. The most stable isomers of ${\text{P}}_{29}^{+}$ and ${\text{P}}_{31}^{+}$ are characterized by single P-P bonds bridging units inside the clusters. Further analysis shows that the pnicogen bonds play an important role in the stabilization of these clusters. These results show that the new developed program is effective and robust in searching global minimum structures for atom clusters, and it also provides new insights into the role of pnicogen bonds in phosphorus clusters.

## Introduction

Clusters bridge atoms, molecules and bulk matter (Johnstom, 2002; Castleman and Jena, 2006; Fehlner et al., 2007; Ha et al., 2019), showing their great potentials for applications in many research fields such as catalysis (Liu and Corma, 2018; Du et al., 2020) and energy storage (VanGelder et al., 2018; VanGelder et al., 2019). They are also characterized by their geometries and electronic structures in many cases (Johnstom, 2002; Fehlner et al., 2007; Ferrando, 2015; Luo et al., 2016; Jena and Sun, 2018; Ha et al., 2019). There are many wonderful examples, including the cage-like fused-ring structure (truncated icosahedron) of C_60_ fullerene (Kroto et al., 1985), the tetrahedral structure of Au_20_ (Li et al., 2003), C_6v_ symmetry boron cluster of B_36_ (Piazza et al., 2014), the borospherene cluster of B_40_ (Zhai et al., 2014; Li et al., 2017), and the protonated serine octamer (Cooks et al., 2001; Kong et al., 2006; Scutelnic et al., 2018). In lots of cases, the structural information of the clusters can hardly be obtained directly from experiments, and theoretical calculations are very important to provide structural candidates whose predicted properties should be further compared with the experimental results, in order to make the identification stable (Zhang and Glezakou, 2020).

Although the structural determination of small molecules based on density functional theory (DFT) or other methods has become a relatively routine task for computational chemists, the identification of the global minimum structures for clusters, especially those with large sizes, is still a great challenge. The reason is that the complexity of the searching space in their potential energy surface (PES) grows exponentially with the increasing number of atoms inside the clusters. Since the numbers of local minima grow quickly with the size of clusters, the global optimization becomes a very difficult task to overcome. Thus, different search algorithms and methodologies, including the genetic algorithm (GA) (Hartke, 1993; Deaven and Ho, 1995; Hartke, 1995; Daven et al., 1996; Rogan et al., 2013; Kanters and Donald, 2014; Shayeghi et al., 2015; Lazauskas et al., 2017; Rabanal-León et al., 2018; Jäger et al., 2019; Yañez et al., 2019) and relative evolutionary algorithm (EA) (Zhou et al., 2020), the swarm intelligence algorithm (Wang et al., 2012; Jana et al., 2019) and others, have been proposed and applied in the past decades (Zhang and Glezakou, 2020).

It is now accepted that the choosing of a suitable method for a special system based on its properties is very important. Usually, for clusters with tens of atoms, a very detailed investigation on their potential energy surfaces is still too difficult to be performed with reasonable computational cost. The Basin-Hopping (BH) algorithm, has been suggested as a good choice to solve global minima of Lennard–Jones clusters (Wales and Doye, 1997; Wales and Scheraga, 1999). Programs based on the BH algorithm have been developed based on empirical potentials (Zhan et al., 2005; Paz-Borbón et al., 2007) and DFT methods (Yoo et al., 2004.; Bai et al., 2006; Bulusu and Zeng, 2006; Huang et al., 2010; Jiang et al., 2012; Chen et al., 2017; Zhao et al., 2017; Chen et al., 2019). And the self-consistent charge density functional tight binding (SCC-DFTB) methods, including DFTB2, DFTB2-γh, DFTB2-γh + gaus and others, have been also applied (Choi et al., 2013).

Considering the success of the method of GFN-xTB, which was a DFTB3 variant developed by Grimme et al. (2017) (Bannwarth et al., 2019), the current work presents a new BH program named NKCS based on the Python, in conjunction with the xTB method for searching global minima of atomic clusters. Phosphorus clusters are selected to be studied, due to the two facts. The first one is that phosphorus exhibits a variety of structural phases, such as orthorhombic black, rhombohedral, violet, metallic, fibrous red, white, and amorphous. Thus, a better understanding about phosphorus clusters can deepen our knowledge about its structures and properties. The second fact is that although phosphorus cluster ions with wide size distributions have been observed in the laser ablation experiments for a long time (Martin, 1986; Mu et al., 2015; Yang et al., 2016; Kong et al., 2019), its structural studies are still limited for small to medium-sized clusters (Guo et al., 2004; Xue et al., 2010; Mu et al., 2015; Kong et al., 2019). And searching for the global minima in their PES is still a challenging task due to the diverse bonding patterns of the element.

## Methods

**FIGURE 1:**
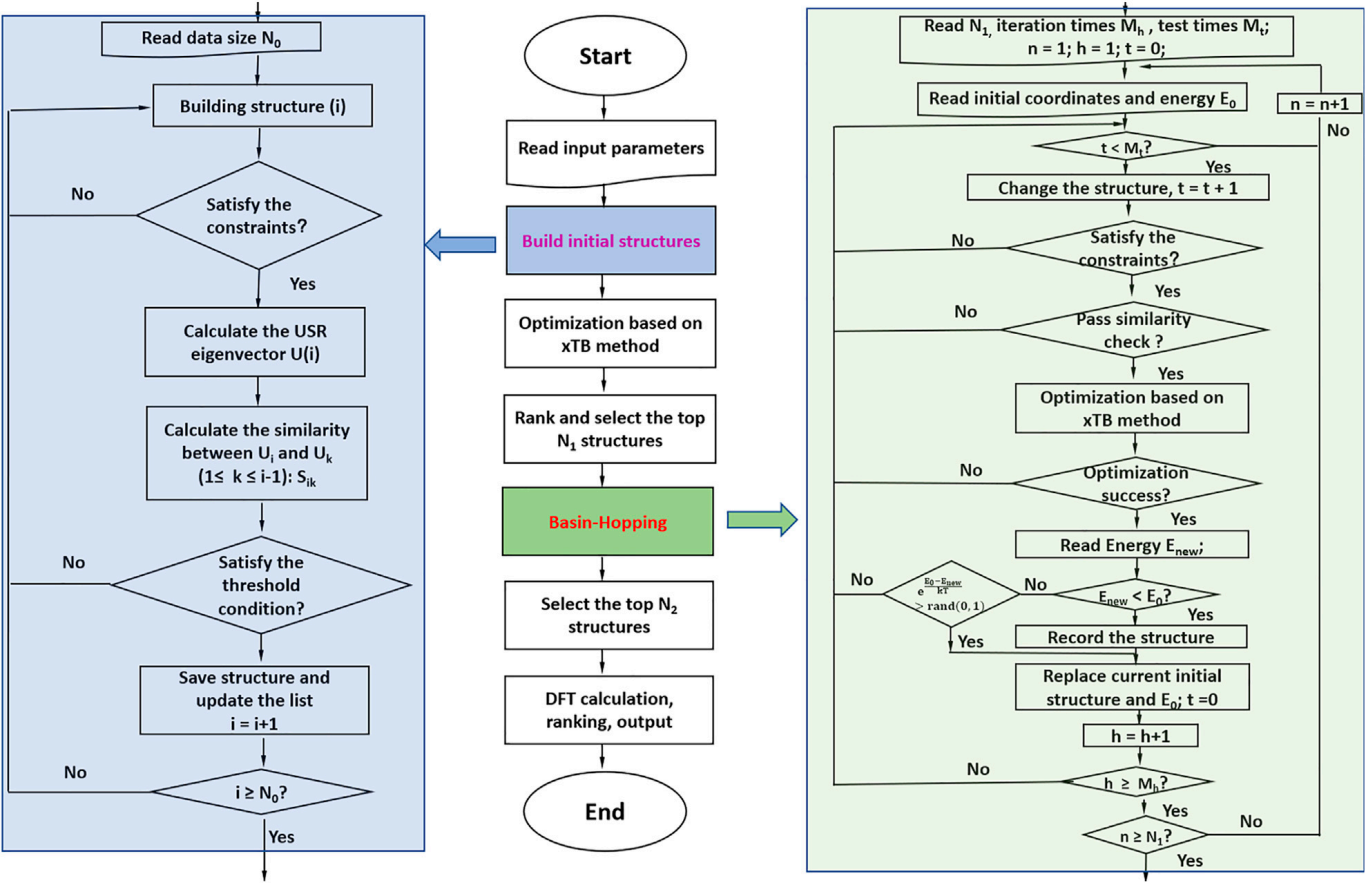
The procedure of the whole program is shown in the middle. The left side shows the process of building initial structures, and the right side shows the detail of the basin-hopping algorithm applied here.

The newly developed program NKCS described here is written in Python language. The procedure of the program is shown in Figure 1. It couples the xTB-based local optimization and BH global search algorithm. For all initially generated or distorted structures through the BH processes, xTB-based local optimization was applied. Then these structures were ranked according to their energies. In the last step, the selected isomers were further sent to high-level DFT calculations. The procedure of the program is shown in the middle part of the picture. According to the input parameters set by the user, initial structures of the clusters are randomly built for xTB optimization. Then the BH algorithm are employed to some selected isomers and during the process, the criteria to accept newly distorted isomers are judged according to their energies calculated by the xTB method. At last, some local minima are further selected to perform high-level DFT calculations to identify the global minimum.

To improve the efficiency of the program, the randomly generated or distorted geometries should be instantly checked according to their inter-atomic distances. Unreasonable structures with inter-atomic distances much smaller than the sum of their covalent radii are directly discarded. After that, a similarity check algorithm is applied to avoid duplicated structures. In this process, the ultrafast shape recognition (USR) algorithm is applied to compare the similarities of the randomly generated or distorted structures and the ones stored in the database (Ballester and Richards, 2007a; Ballester and Richards, 2007b; Ballester et al., 2009). For the homo atomic clusters studied here, the previously suggested 12 descriptors were applied here. The set of intra-cluster atomic distances from four locations are considered: the molecular centroid (*ctd*), the closest atom to *ctd* (*cst*), the farthest atom from *ctd* (*fct*) and the farthest atom from *fct* (*ftf*). So a molecule can be described as: $\vec{M}=\left(\mu_{1}^{ctd},\mu_{2}^{ctd},\mu_{3}^{ctd},\mu_{1}^{cst},\mu_{2}^{cst},\mu_{3}^{cst},\mu_{1}^{fct}\;\mu_{2}^{fct},\mu_{3}^{fct},\mu_{1}^{ftf},\mu_{2}^{ftf},\mu_{3}^{ftf}\right)$ *.* In this way, each structure can be described by the 12 numbers, and the similarity of two structures *i* and *k* can be calculated as:

$$S_{ik}=\frac{1}{\left(1+\frac{1}{12}\sum_{l=1}^{12}\left(\left|M_{l}^{i}-M_{l}^{k}\right|\right)\right)}$$

where $M_{l}^{i}$, $M_{l}^{k}$ are the *l*th USR descriptors of the *i*th and *k*th structures, respectively. The value of *S*_*ik*_ is limited between 0 and 1. A high value of *S*_*ik*_ indicates that the two isomers have close geometries, and a threshold can be selected in the program to distinguish two structures.

For the randomly constructed structure sets, the xTB method was applied to perform structural optimization and energy calculation. The parametrization in the method covers all spd-block elements and the lanthanides up to Z = 86 and it has been considered as a suitable method for dealing with various clusters with complex electronic structures (Grimme et al., 2017). The BH algorithm was then applied for the selected isomers based on their energies after the xTB optimization. Considering the BH method is one of the individual-based methods (Zhang and Glezakou, 2020), an initialized population with suitable size is applied here to improve its performance in the global optimization. After the selection of initial seed structures for BH algorithm based on their energies calculated by the xTB method, distorted structures are generated from the seeds by the displacement. The reasonability and similarity of the new structure should be checked and then optimized by the xTB method. For the acceptance of the newly distorted structure, the previously suggested criteria by Zhou et al. are applied here (Zhou et al., 2019). And the NKCS program also integrates the interface of Gaussian computing software (Frisch et al., 2009) to perform high-level structure optimization and frequency calculation for the selected isomers by the BH algorithm.

## Results and Discussion

**FIGURE 2:**
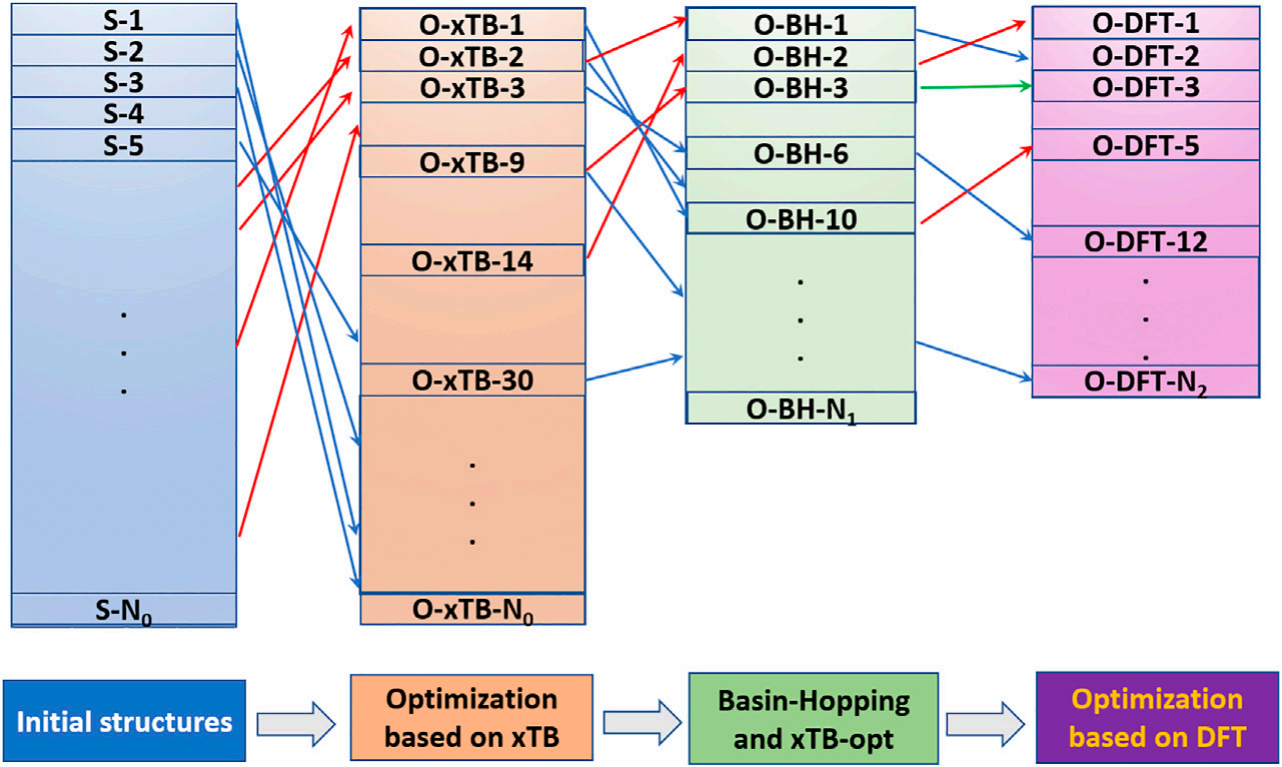
The process for searching the global minimum by the NKCS program.

The NKCS program has been tested with the odd-numbered phosphorus cluster cations here. In order to make the process clear, an example for searching the global minimum of ${\text{P}}_{15}^{+}$ is displayed in Figure 2. The initial population size for clusters (N_0_) with N atoms is set as ∼ N^2.8^ for all the phosphorus clusters studied here. For ${\text{P}}_{15}^{+}$, after the reasonability and similarity check, 2000 structures were generated randomly. The distance between two adjacent phosphorus atoms is limited between 2 and 3 Å, and the USR threshold was selected to be 0.98. These 2000 structures were optimized by the xTB method and the top N_1_ (30 in this case) structures were selected as seeds for BH processes. During the process, structural check for the new distorted isomers were also performed. If a new structure is more favorable in energy than the seed, it will be recorded and accepted as the new seed. Otherwise, the probability of accepting the structure, $e^{\frac{E_{0}-E_{new}}{kT}}$ , would be compared with a random number located in (0,1) to decide whether it should be taken as a new seed. For all the phosphorus cluster cations here, the temperature *T* was set at 273 K. After the BH process, the top N_2_ (30 in this case) structures were further selected for DFT calculation. These isomers would be optimized and ranked at the level of B3LYP/6–311+G(d) by the Gaussian 09 program.

**FIGURE 3:**
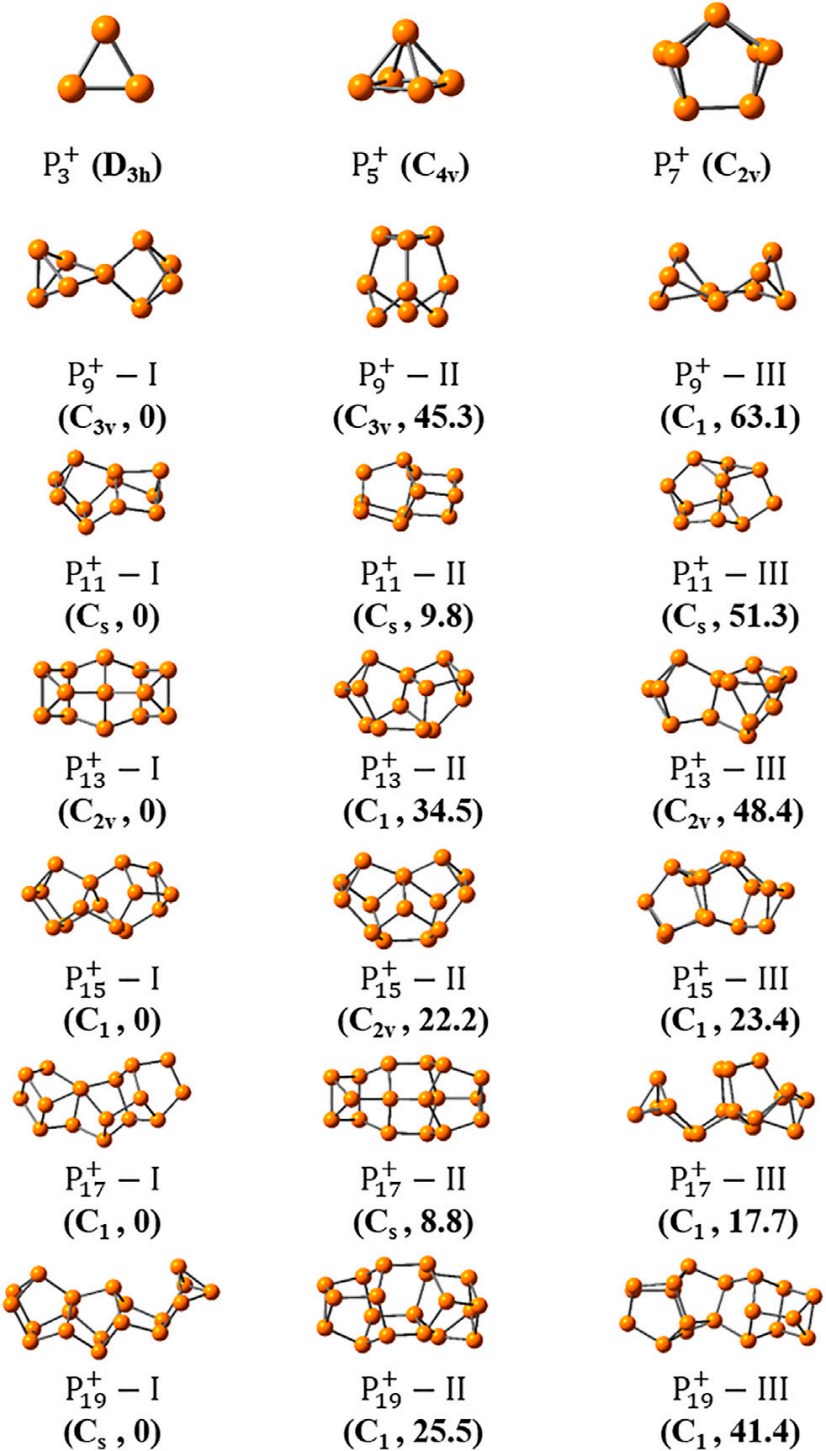
The most stable isomers of ${\text{ P}}_{2\text{n}+1}^{+}$ (*n* = 1–9) searched by the NKCS program. The three most stable isomers are identified as ${\text{P}}_{2\text{n}+1}^{+}-\text{I}$, ${\text{P}}_{2\text{n}+1}^{+}-\text{II}$, and ${\text{P}}_{2\text{n}+1}^{+}-\text{III}$, in turn. Their symmetries and relative energies (in kJ/mol) to corresponding global minima are shown below.

**FIGURE 4:**
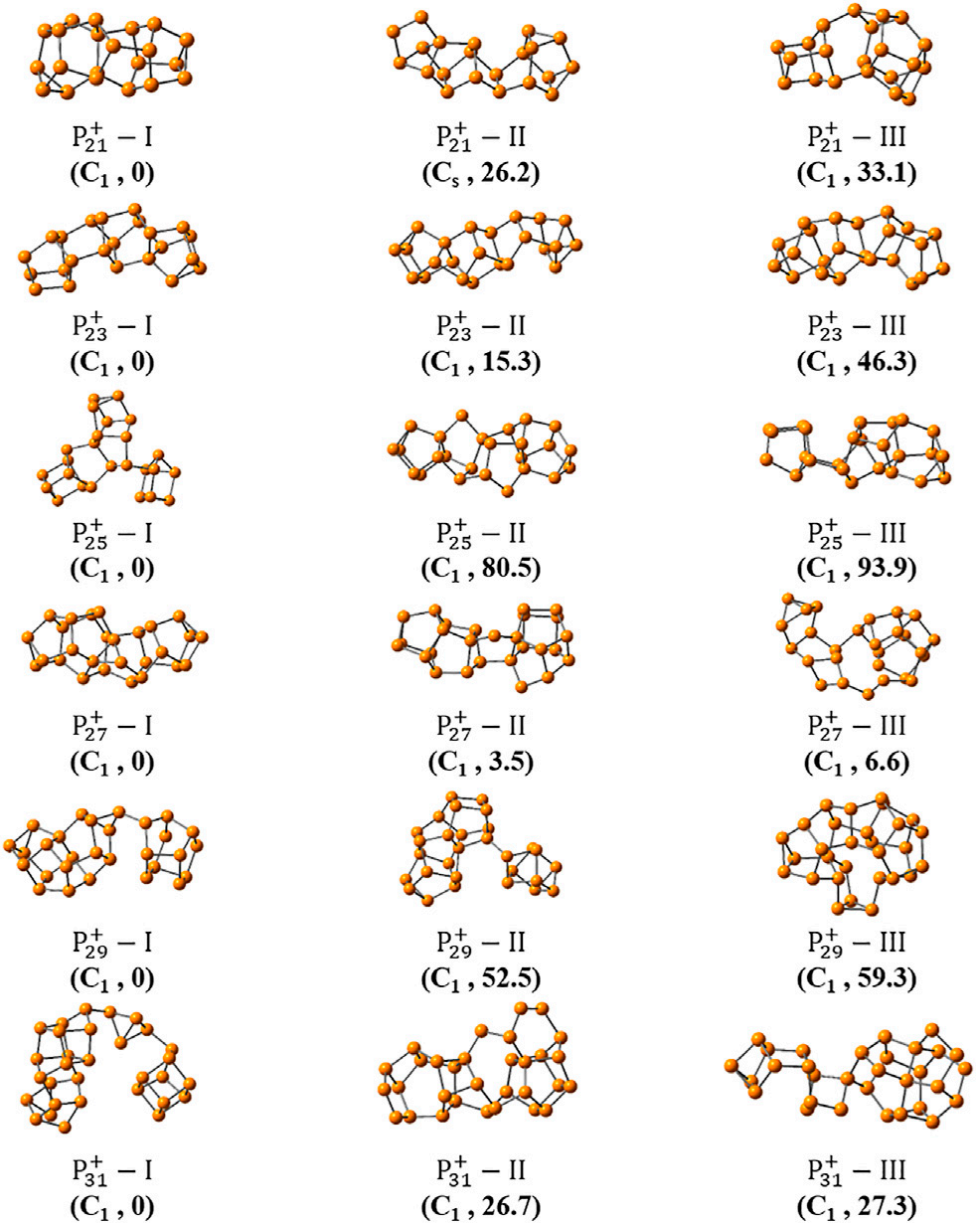
The three most stable isomers of ${\text{ P}}_{2\text{n}+1}^{+}$ (*n* = 10–15) searched by the NKCS program. Their symmetries and relative energies (in kJ/mol) to corresponding global minima are shown below.

Figures 3, 4 show the optimized global minimum geometries of ${\text{P}}_{2n+1}^{+}$ (*n* = 1 ∼ 15). To make sure that the results obtained are indeed global minima, the program has been run three times for each phosphorus cluster. For all reported cationic phosphorus clusters, frequency calculations are preformed to ensure that they are true minima on the potential energy surfaces. For small cationic clusters of ${\text{P}}_{3}^{+}$, ${\text{P}}_{5}^{+}$ and ${\text{P}}_{7}^{+}$, the most stable isomers with D_3h_, C_4v_ and C_2v_ symmetries in turn, have been revealed by Guo et al. (2004), Xue et al. (2010) previously. These structures have also been reproduced here and are shown in Figure 3. For ${\text{P}}_{9}^{+}$, the previously suggested lowest-energy geometry with D_2d_ symmetry was reproduced too (Xue et al., 2010). The second stable isomer of ${\text{P}}_{9}^{+}-\text{II}$, has an energy 45.3 kJ/mol higher than that of the former one. It consists of a P_6_ unit with a chair-like structure below and a triangle of P_3_ unit above, characterized by its C_3v_ symmetry. For ${\text{P}}_{11}^{+}$, besides the previously reported structure (${\text{P}}_{11}^{+}-\text{II}$) (Xue et al., 2010), a new isomer of ${\text{P}}_{11}^{+}-\text{I}$ with a C_s_ symmetry, was found to be more stable by 9.8 kJ/mol than the former. For clusters with larger sizes, more stable isomers were found. To make them clear, the top eight isomers of ${\text{P}}_{2n+1}^{+}$ (*n* = 6–15) were all shown in Supplementary Figures S1, S2. For ${\text{P}}_{13}^{+},$ the previously suggested most stable isomer (${\text{P}}_{13}^{+}-\text{II})$ was found to be accompanied with a more stable isomer, ${\text{P}}_{13}^{+}-\text{I}$. The latter is characterized by its C_2v_ symmetry and has an energy 34.5 kJ/mol lower than that of ${\text{P}}_{13}^{+}-\text{II}$. For ${\text{P}}_{15}^{+}$, the found most stable isomer is the same as the one reported by Xue et al. (2020) previously. Another isomer of ${\text{P}}_{15}^{+}-\text{II}$ with C_2v_ symmetry was also found by the program, which has an energy 22.2 kJ/mol higher than ${\text{P}}_{15}^{+}-\text{I}$. Interestingly, this isomer ${\text{P}}_{15}^{+}-\text{II}$ can be formed by adding two P atoms in the middle of ${\text{P}}_{13}^{+}-\text{II}$. For ions of ${\text{P}}_{17}^{+}$, the previously reported isomer was found as the 10th most stable isomer (Xue et al., 2010). Nine more stable isomers have been identified and the three most stable isomers of ${\text{P}}_{17}^{+}-\text{I},\text{II}$ and III are shown in Figure 3. The isomer ${\text{P}}_{17}^{+}-\text{I}$, which has an energy 59.6 kJ/mol lower than the previously reported one, can be regarded as a P_8_ cuneate unit connected with a P_7_ norbornane though a P_2_ unit, which has no symmetry. For ${\text{P}}_{19}^{+}$, 20 new isomers were found to have lower energies than the one previously reported. The top three isomers are shown in Figure 3, in which the most stable one has an energy 140.9 kJ/mol lower than the one reported before. And it is also characterized by a plane of symmetry.

As the size increased, the symmetry of the cluster ions decreases. The newly found most stable isomer of ${\text{P}}_{21}^{+}$ (${\text{P}}_{21}^{+}-\text{I}$), is more energy favorable than the previously reported one by 84.3 kj/mol. For ${\text{P}}_{23}^{+}$, the discovered isomer of ${\text{P}}_{23}^{+}-\text{I}$ has an energy 15.3 kJ/mol lower than the previously reported isomer of ${\text{P}}_{23}^{+}-\text{II}$ (Xue et al., 2010). Unlike other cluster ions of ${\text{P}}_{2\text{n}+1}^{+}$ (*n* = 7–11), the new isomer of ${\text{P}}_{25}^{+}-\text{I}$ does not has the chain-like geometry. There is a P_6_ trigonal prism unit on the right side, suspended in the middle of the long P_18_ unit on the left by a single bond. This isomer is energetically more preferred by 80.5 kJ/mol than the previously suggested one (${\text{P}}_{25}^{+}-\text{II}$ in Figure 4).

And the most stable isomers of ${\text{P}}_{27}^{+}$, ${\text{P}}_{29}^{+}$ and ${\text{P}}_{31}^{+}$ were suggested here for the first time. These structures are more complicated and show no symmetry (Figure 4). The most stable isomer of ${\text{P}}_{27}^{+}-\text{I}$ has a typical linear structure that likes that of ${\text{P}}_{23}^{+}-\text{I}$ or ${\text{P}}_{25}^{+}-\text{II}$. The structure of ${\text{P}}_{29}^{+}-\text{I}$, can be regarded as that its left and right sides are connected by a single P-P bond. The left side of the cluster ion has a compact unit of P_16_, and right side has a unit of P_11_ that can be regarded as a six-member ring connected with a five-member ring directly. For the second stable isomer ${\text{P}}_{29}^{+}-\text{II}$, the two units of P_20_ and P_9_ are also linked by a single bond. Interestingly, the structure of ${\text{P}}_{31}^{+}-\text{I}$ includes three parts of P_19,_ P_4_, and P_9_, in which the first and the latter two parts are both connected though P-P bonds, respectively. And the whole ion has a curved linear structure. In order to make the results more reliable, calculation based on the level of MP2/6–311+G(d)//B3LYP/6–311+G(d) were performed for the top three isomers of ${\text{P}}_{2\text{n}+1}^{+}$ (*n* = 12–15). Although the values of their relative energies are some different, their orders in energies keep unchanged (Supplementary Table S1 in the supporting information).

Briefly, the BH algorithm based on the xTB method has been developed for searching global minima of clusters. Considering the parametrization of xTB method covers all spd-block elements and the functional form of the xTB mostly avoids element-pair-specific parameters, the program developed here has a very wide range of applications. And compared with DFT-based methods, it significantly saves computing time. Medium-sized phosphorus cluster cations were studied here, and new energetically favored structures were identified. Based on these results, some structural rules of these cationic clusters should be further discussed, since it might be helpful to get some general pictures about these energetically preferred structures and structural tendency about large-sized clusters.

For one thing, it is interesting to find that symmetric structures are very important for small-sized clusters of ${\text{P}}_{2\text{n}+1}^{+}$ (*n* = 1–6). For ${\text{P}}_{19}^{+}$, the most stable isomer also has a C_s_ symmetry. For ${\text{P}}_{15}^{+},{\text{P}}_{17}^{+}$, and ${\text{P}}_{21}^{+}$, although none of their most stable isomers have symmetry, their second most stable isomers have C_2v_, C_s_, and C_s_ symmetry, in turn. For clusters with larger size of ${\text{P}}_{2\text{n}+1}^{+}$ (*n* = 11–15), all their top three stable isomers show no symmetry. Although this, the symmetry of local unit in the large-size clusters still exists. For example, both units in ${\text{P}}_{29}^{+}-\text{I}$ linked by a single bond have rough C_s_ symmetry. These results also suggest the importance of an unbiased method in searching the global minima of large-sized clusters, which can cannot be directly replaced by simple intuitions.

**FIGURE 5:**
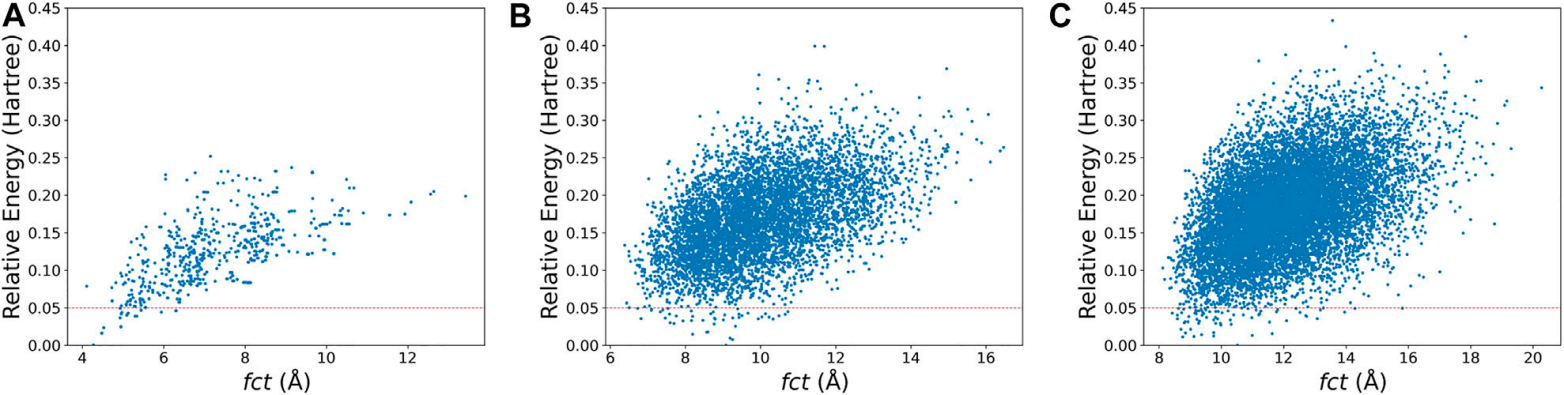
The relative energy-*fct* diagrams for the xTB-optimized structures of the randomly generated populations of **(A)** ${\text{P}}_{9}^{+},$ **(B)** ${\text{P}}_{19}^{+}$ and **(C)** ${\text{P}}_{31}^{+}$. Every dot in the picture corresponds one structure and the dots below the red line indicates the isomers have relative energies less than 0.05 hartree compared to the corresponding global minimum in each subgraph.

For the second point, most medium-sized globe minima of ${\text{P}}_{2\text{n}+1}^{+}$ (*n* = 5–15) exhibit chain-like configurations, expect that of ${\text{P}}_{25}^{+}$. And the common building units include P_7_, P_8_, and P_9_ building blocks. For clusters with larger sizes, the chains become curved. A statistical view on the size of the energetically preferred structures may provide some clues. The USR parameter *ftf* that indicates the distance between the farthest atom from *fct* can be applied as an indicator of the length of the cluster. Based on the xTB calculation results of the initial population built up in the second step of Figure 2, a general picture descripting the relationship between energy and length can be obtained. Figure 5 shows the relative energy-*fct* diagrams for the xTB-optimized structures of the randomly generated populations of ${\text{P}}_{9}^{+},$ ${\text{P}}_{19}^{+}$, and ${\text{P}}_{31}^{+}$. It can be found that the most of energetically preferred structures for ${\text{P}}_{9}^{+}$ have a distribution of *fct* in the range of 4–6 Å. For ${\text{P}}_{19}^{+}$ and ${\text{P}}_{31}^{+}$, the lengths of energetically preferred structures were concentrated in the range of 7–10 and 8–12 Å, respectively. The results indicate that the lengths of the clusters grow with their sizes, but not linearly, and the curved structures will be more general for large-size clusters.

**FIGURE 6:**
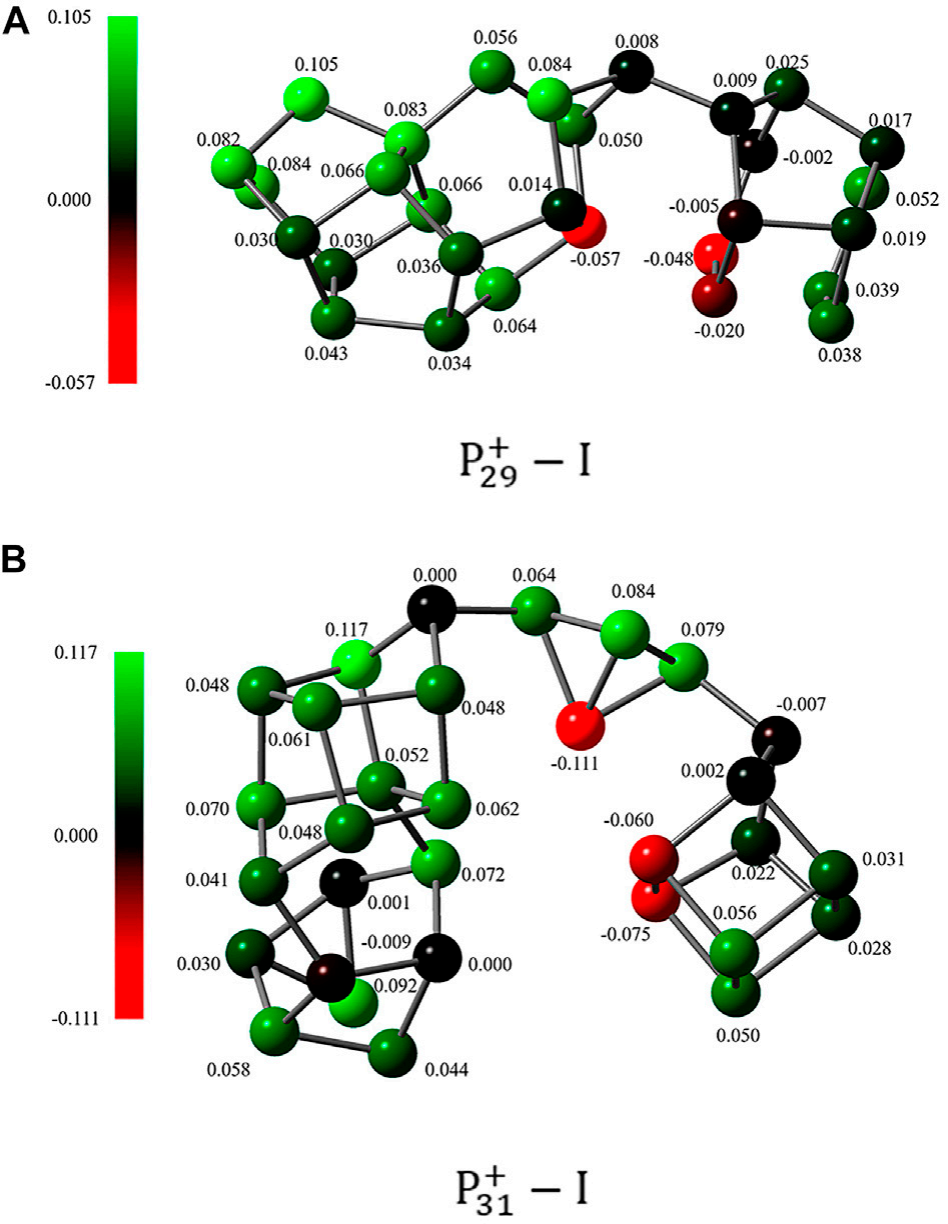
The NBO charge distribution of **(A)** ${\text{P}}_{29}^{+}-\text{I}$ and **(B)** ${\text{P}}_{31}^{+}-\text{I}$.

For the third point, it is interesting to found that both isomers of ${\text{P}}_{29}^{+}-\text{I}$ and ${\text{P}}_{31}^{+}-\text{I}$ are characterized by single P-P bonds bridging units inside the clusters. Although this kind of bridging bonds is reasonable in forming one- or two-dimensional phosphorus nanomaterials, it is lesser-known for middle- or large-size homoatomic clusters. For homoatomic clusters, it is usually to suggest that the clusters are inclined to take compact structures with high symmetry or consistent linear structures. So why these clusters are so different? A possible explanation is that the weak polarities of these intra-cluster covalent bonds are distributed in a way to stabilize the whole cluster by enhancing their charge-charge, charge-dipole, or dipole-dipole interactions. However, the natural bond orbital (NBO) charge distribution (Carpenter and Weinhold, 1988; Reed et al., 1988) of these clusters shows although this interaction may help to stabilize the isomer in some extent, it should not be the main reason (Figure 6).

**FIGURE 7:**
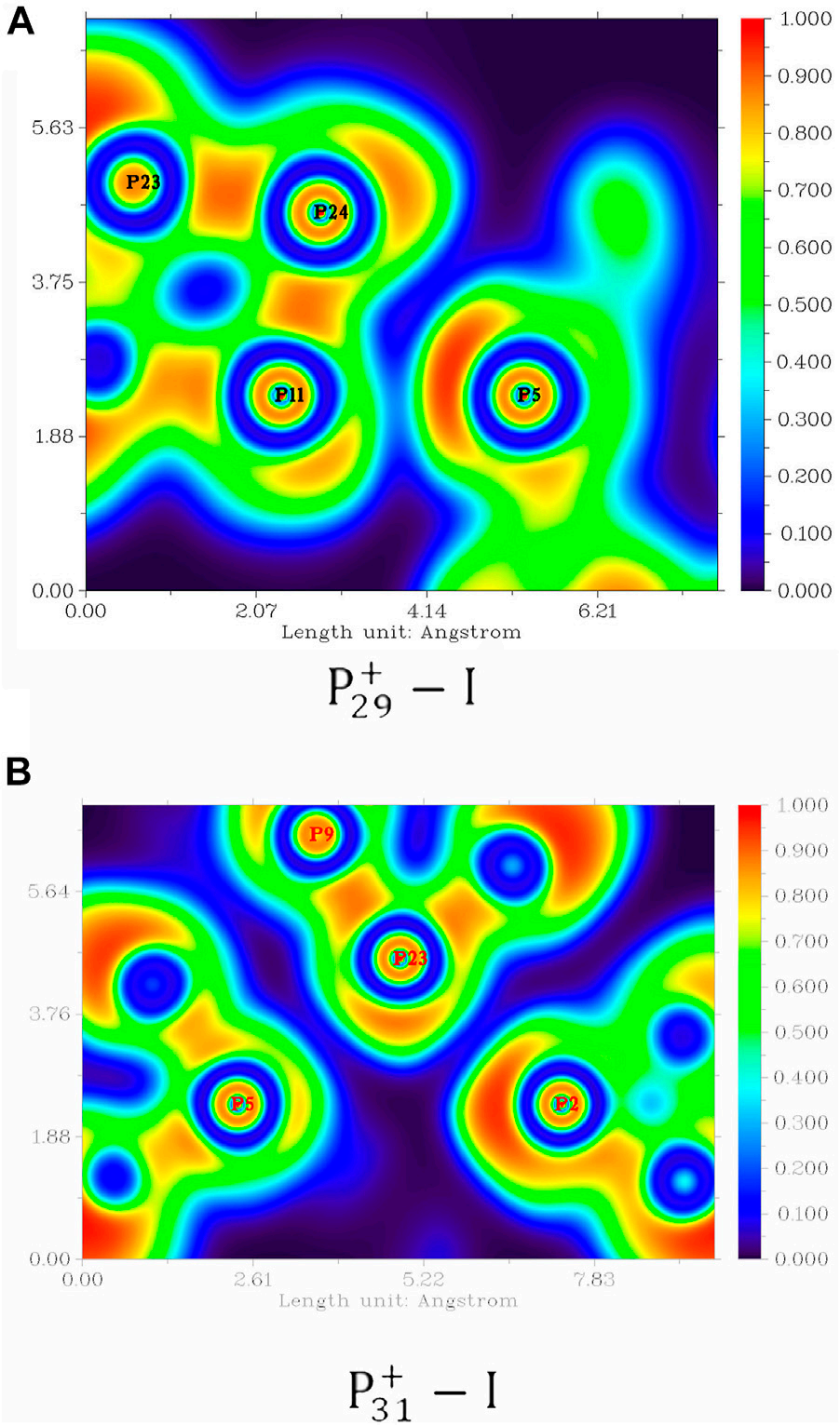
ELFs of **(A)** ${\text{P}}_{29}^{+}-\text{I}$ and **(B)** ${\text{P}}_{31}^{+}-\text{I}$.

**FIGURE 8:**
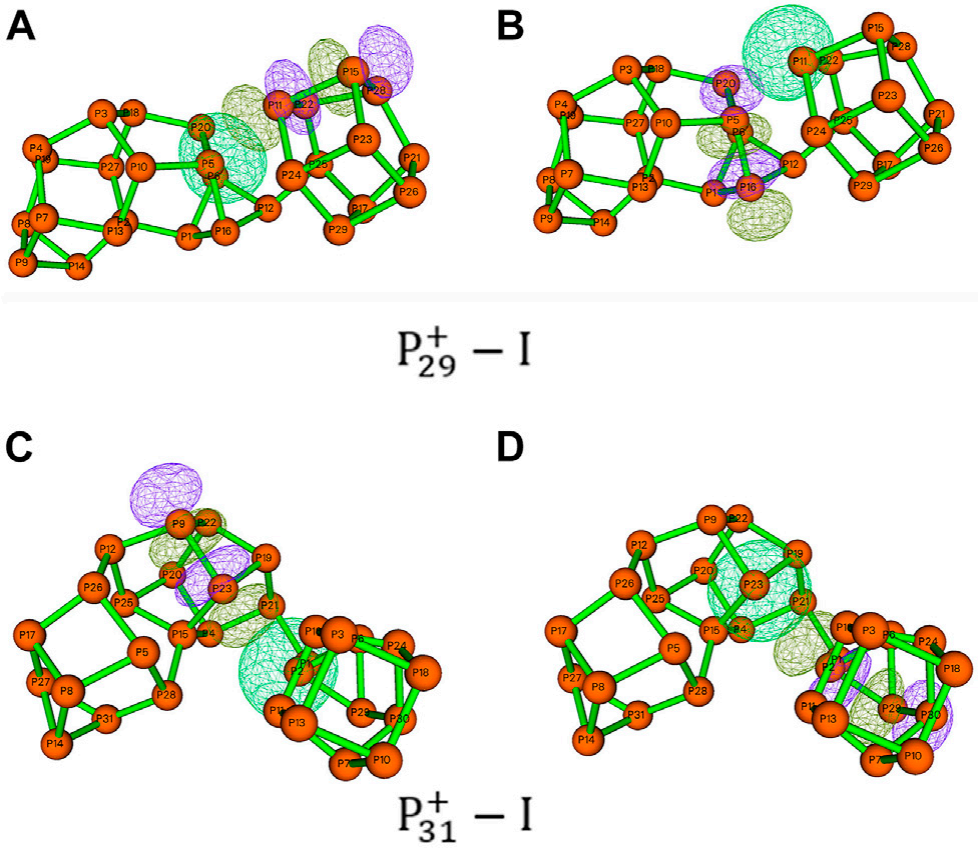
Hyperconjugation of LP(P) → σ*(P–P). The natural bonding orbits were obtained by NBO analysis of **(A,B)** ${\text{P}}_{29}^{+}-\text{I}$ and **(C,D)** ${\text{P}}_{31}^{+}-\text{I}$.

On the other hand, the short distances between the nonbonding phosphorus atoms indicate that the pnicogen bonds (Zahn, et al., 2011; Scheiner, 2013) inside the clusters may play a very important role. In ${\text{P}}_{29}^{+}-\text{I}$, the distances of P5···P11 is 294 pm, which is below the sum of their van der Waals radii of 380 pm. Similarly, the distances of P2···P23 in ${\text{P}}_{31}^{+}-\text{I}$ is 333 pm. These attractive P···P interactions are very similar to those previously reported pnicogen bonds in carbaboranes. Chemical bonding analyses were also examined by electron localization function (ELF) analysis with the program of Multiwfn (Lu and Chen, 2012). As shown in Figure 7, regions between P11 and P5 in ${\text{P}}_{29}^{+}-\text{I}$, and between P2 and P23 in ${\text{P}}_{31}^{+}-\text{I}$, are both characterized by their electron-pair densities. The pnicogen interaction can be further investigated by the second-order perturbation approach. As the example of ${\text{P}}_{29}^{+}-\text{I}$ shown in Figure 8A and B, the interactions of LP(P5) →σ*(P11-P15) and LP(P11) →σ*(P5-P16) in ${\text{P}}_{29}^{+}-\text{I}$ have the stabilization energies of 9.34 and 3.70 kcal/mol, respectively, showing a very strong pnicogen bond. The second example of ${\text{P}}_{31}^{+}-\text{I}$ is shown in Figure 8C and D. The hyperconjugation of the lone pair of electrons at P2 with the adjacent phosphorus–phosphorus bond P23-P9 (LP(P2) →σ*(P23-P9)) was observed with a second-order perturbation stabilization energy of 2.66 kcal/mol. At the same time, the interaction of LP(P23) →σ*(P2-P29) also contributes 0.87 kcal/mol in stabilization the pnicogen bond.

Both Wiberg bond index (WBI) and QTAIM topological analysis were employed to analyze the bonding nature of these bonds. The calculated total WBI values of the bonds P5···P11 in ${\text{P}}_{29}^{+}-\text{I}$, P2···P23 in ${\text{P}}_{31}^{+}-\text{I}$ are 0.14 and 0.04, respectively, supporting the existence of the pnicogen bonds. Table 1;Supplementary Table S2, show the results of AIM topology parameters, including electron density (*ρ*_*e*_) at the pnicogen bond critical points and Laplacian (▽^2^ *ρ*_*e*_). The results suggest that intracluster pnicogen bonds play a very important role for their structural stabilization and isomerization. It is also found that the pnicogen bond is important for other curved clusters with small sizes (shown in Figure 4). For example, the structure of ${\text{P}}_{27}^{+}-\text{I}$ is characterized by a strong pnicogen bond with a P1 … P4 distance of 297 pm (with a WBI of 0.26). The NBO and QTAIM analysis also support the interaction (Supplementary Figures S3, S4; Table 1 and Supplementary Table S2).

**TABLE 1:** Electron densities (*ρ*_*e*_, a.u.), Laplacian of the electron densities (▽^2^ *ρ*_*e*_, a.u.), Pnicogen bond distances (Å), and Wiberg Bond Indexes (WBI) of the pnicogen bonds in ${\text{P}}_{27}^{+}-\text{I },{\text{ P}}_{29}^{+}-{\text{I and P}}_{31}^{+}-\text{I}$, compared with those of some pnicogen bonds previously reported.^a^

Bonds	*ρ*_*e*_	▽^2^ *ρ*_*e*_	Pnicogen bond distances (Å)	WBI	Ref
P1 … P4 (${\text{P}}_{27}^{+}-\text{I}$)	0.0258	0.0415	2.97	0.26	This study
P5 … P11 (${\text{P}}_{29}^{+}-\text{I}$)	0.0280	0.0419	2.94	0.14	This study
P2 … P23 (${\text{P}}_{31}^{+}-\text{I}$)	0.0137	0.0282	3.33	0.04	This study
P … P (PH_2_Cl···PCl_3_)	–	–	3.40	0.04	Zahn et al. (2011)
P … P (PH_2_Cl···PH_2_F)	–	–	2.99	0.12	Zahn et al. (2011)
P … N (FH_2_P···NCCl)	0.0170	0.0590	2.80	0.04	Esrafili and Mousavian (2018)
P … N (ClH_2_P···NCH)	0.0126	0.0513	2.89	–	Esrafili and Sadr-Mousavi (2017)
P … N (ClH_2_P···NCH···C_2_H_2_)	0.0133	0.0468	2.87	–	Esrafili and Sadr-Mousavi (2017)
P … Bi (structure **2a**)	0.0131	0.0267	3.58(T), 3.37 (E)^b^	0.09	Mokrai and Benko (2019)
P … Cl (PH_3_-BrCl)	0.0051	0.0176	3.68	–	Wu et al. (2019)
P … Cl (PH_2_F-BrCl)	0.0091	0.0316	3.26	–	Wu et al. (2019)
P … Cl (FCl···PH_3_···NCH)	–	–	2.22	0.78	Del Bene et al. (2017)

a The AIM topology analysis of pnicogen bonds reported here is performed using Multiwfn program (Lu and Chen, 2012), while other results were taken from references directly.

b T and E indicate theoretical and experimental values, respectively.

Although pnicogen bonds have been previously reported and studied for different species by many research groups, this is still the first study showing that the pnicogen bonds can exist and play important roles in homoatomic phosphorus clusters without the help of other ligands or heteroatoms. By comparing the pnicogen bonds reported herein with other previously reported pnicogen bonds, the important roles of these interactions in phosphorus clusters can be further reflected. Thus, some typical pnicogen bonds were selected from relative references (Zahn, et al., 2011; Del Bene et al., 2017; Esrafili and Sadr-Mousavi, 2017; Esrafili and Mousavian, 2018; Mokrai, and Benko, 2019; Wu, et al., 2019) and were compared with those bonds reported here. Results were shown in Table 1. The P … P pnicogen bonds reported here have similar bond distances and WBIs with those previously reported (Zahn, et al., 2011) and similar to other type pnicogen bonds including PN, P … Bi and P … Cl interactions. And the bond of P1 … P4 in ${\text{P}}_{27}^{+}-\text{I}$ even has the highest WBI except to the special case of P … Cl in the ternary complex of FCl … PH_3_ … NCH, in which the P … N pnicogen-bond was enhanced by the P … Cl halogen bond through the σ-hole (Del Bene, et al., 2017).

Briefly, these results reflected that the intracluster pnicogen bonds can greatly stabilize the cluster, thus play important roles in large-size phosphorus clusters and phosphorus-related materials. On the other hand, the ELF analysis shown in Figure 7 also indicates the possibility of the existence of multiple pnicogen bonds in large-size phosphorus clusters. And a very interesting topic is how the introduce of heteroatom can affect the pnicogen bonds and their most stable structures. So, we hope the result reported here can attract more researchers to focus on this issue.

## Conclusion

A combined algorithm of BH and xTB to locate global minima in potential energy surface of atomic clusters has been developed here. Several strategies, including the similarity check, are considered in the algorithm. The $P_{2\text{n}+1}^{+}$ cluster cations are selected to be studied using the program due to their structural varieties and complexities. For cluster cations of ${\text{P}}_{2\text{n}+1}^{+}$ (*n* = 1–4) and ${\text{P}}_{15}^{+}$, the program reproduced the lowest-energy structures reported previously. For $P_{2\text{n}+1}^{+}$ (*n* = 5, 6, 8–12), new isomers with energies 10 ∼ 80 kJ/mol lower than those previously reported have been identified on the level of B3LYP/6–311+G(d). The most stable isomers of $P_{2\text{n}+1}^{+}$ (*n* = 13–5) are also reported here. Although symmetric structures dominate the most stable isomers of all small-sized clusters of ${\text{P}}_{2\text{n}+1}^{+}$ (*n* = 1–6), their importance decrease for the clusters of ${\text{P}}_{2\text{n}+1}^{+}$ (*n* = 7–10). And for clusters with larger sizes, no symmetry has been observed for all their top three isomers. The lengths of the clusters grow with their sizes, but only distinct for clusters up to ${\text{P}}_{23}^{+}$. Curved structures with single P-P bonds are found to be important for ${\text{P}}_{29}^{+}$ and ${\text{P}}_{31}^{+}$. Further analysis shows that the pnicogen bonds play important roles in these phosphorus clusters. The results show that the new developed xTB-based BH program is effective and robust in searching global minimum structures for atomic clusters. And for large-size phosphorus clusters, a systemic study for a better understanding about the pnicogen bonds is needed very much.

## Data Availability

The raw data supporting the conclusions of this article will be made available by the authors, without undue reservation.
