# Supplementary material for: Search for Global Minimum Structures of P2n+1+ (n = 1–15) Using xTB-Based Basin-Hopping Algorithm
Source: Front Chem. 2021 Jul 26;9:694156. doi: 10.3389/fchem.2021.694156 (PMC8350033; doi:10.3389/fchem.2021.694156)
Supplement: Supplementary file 1 [file DataSheet1.docx]

Supplementary Material

**Search for Global Minimum Structures of** $\mathbf{P}_{\boldsymbol{2}\boldsymbol{n+1}}^{\boldsymbol{+}}$  **(n = 1 – 15) Using xTB-based Basin-Hopping Algorithm**

**Min Zhou^1,2^, Yicheng Xu^2^, Yongliang Cui^2^, Xianyi Zhang^1*^, Xianglei Kong^1,2*^**

^1^ School of Physics and Electronic Information, Anhui Normal University, Wuhu 241000, China

^2^ The State Key Laboratory and Institute of Elemento-Organic Chemistry, Collage of Chemistry, Nankai University, Tianjin 300071, China

^3^ Collaborative Innovation Center of Chemical Science and Engineering, Nankai University, Tianjin 300071, China

*** Correspondence:**Xianyi Zhang
xyzhang@ahnu.edu.cn

Xianglei Kong
kongxianglei@nankai.edu.cn

# Supplementary Data

**Fig. S1** The most stable isomers of $P_{2n+1}^{+}$ (n=6-10) searched by the NKCS program. The eight most stable isomers are identified as $P_{2n+1}^{+}-I$, $P_{2n+1}^{+}-\mathrm{II}$ …. and $P_{2n+1}^{+}-V\mathrm{III}$, in turn. Their symmetries and relative energies (without ZPE correction, in kJ/mol) to corresponding global minima are shown below.


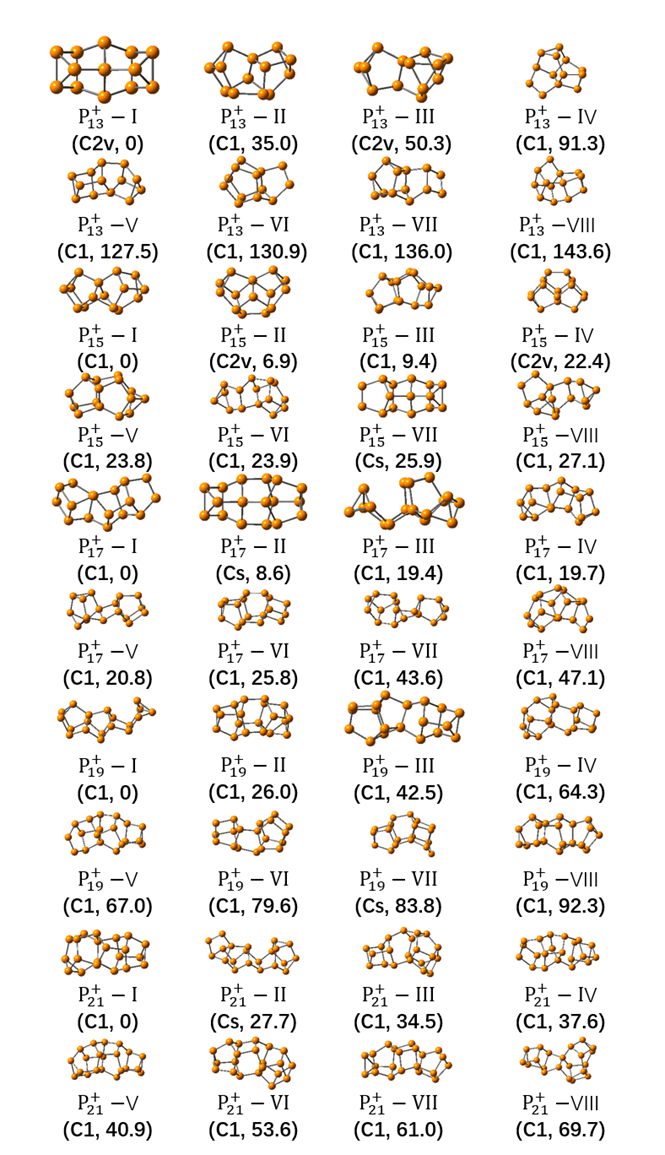


**Fig. S2** The most stable isomers of $P_{2n+1}^{+}$ (n=11-15) searched by the NKCS program. The eight most stable isomers are identified as $P_{2n+1}^{+}-I$, $P_{2n+1}^{+}-\mathrm{II}$ …. and $P_{2n+1}^{+}-V\mathrm{III}$, in turn. Their symmetries and relative energies (without ZPE correction, in kJ/mol) to corresponding global minima are shown below.


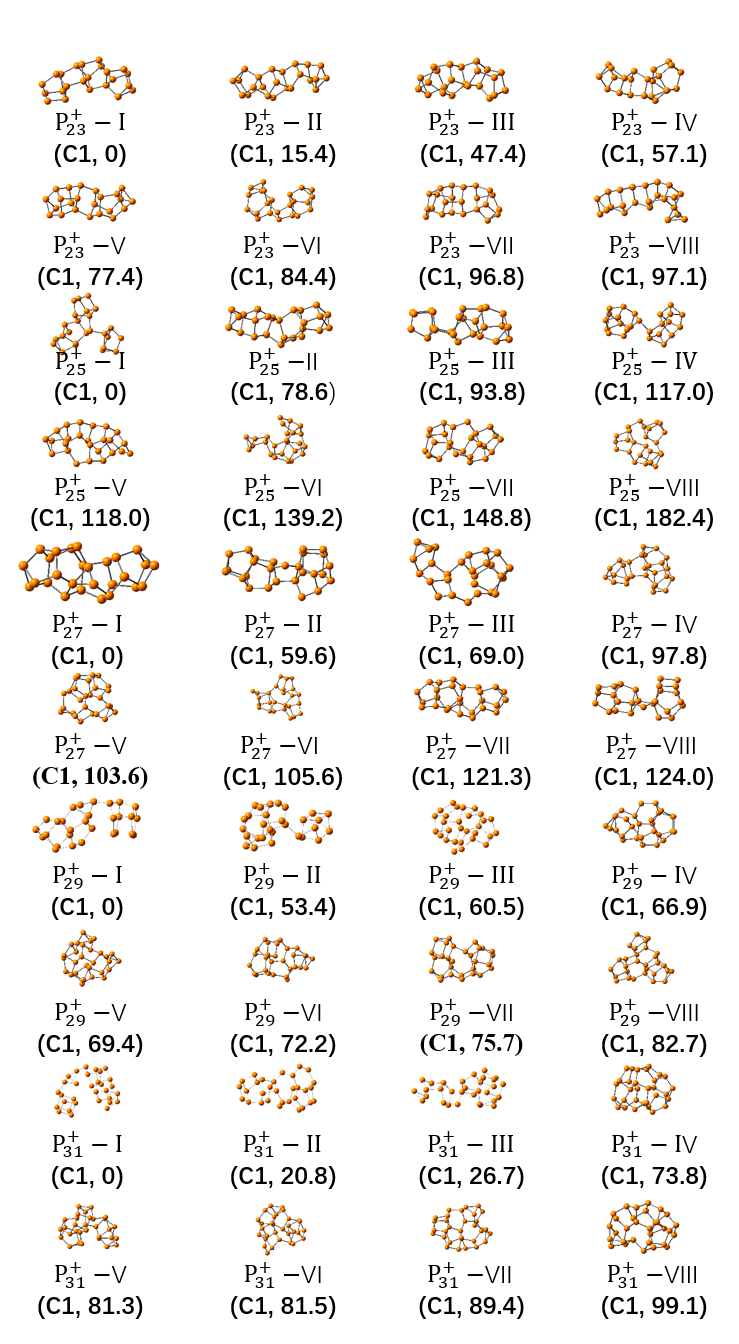


**Fig.S3** The NBO charge distribution, electron localization function (ELF) and natural bonding orbits of $P_{27}^{+}-I$ are listed from top to bottom.


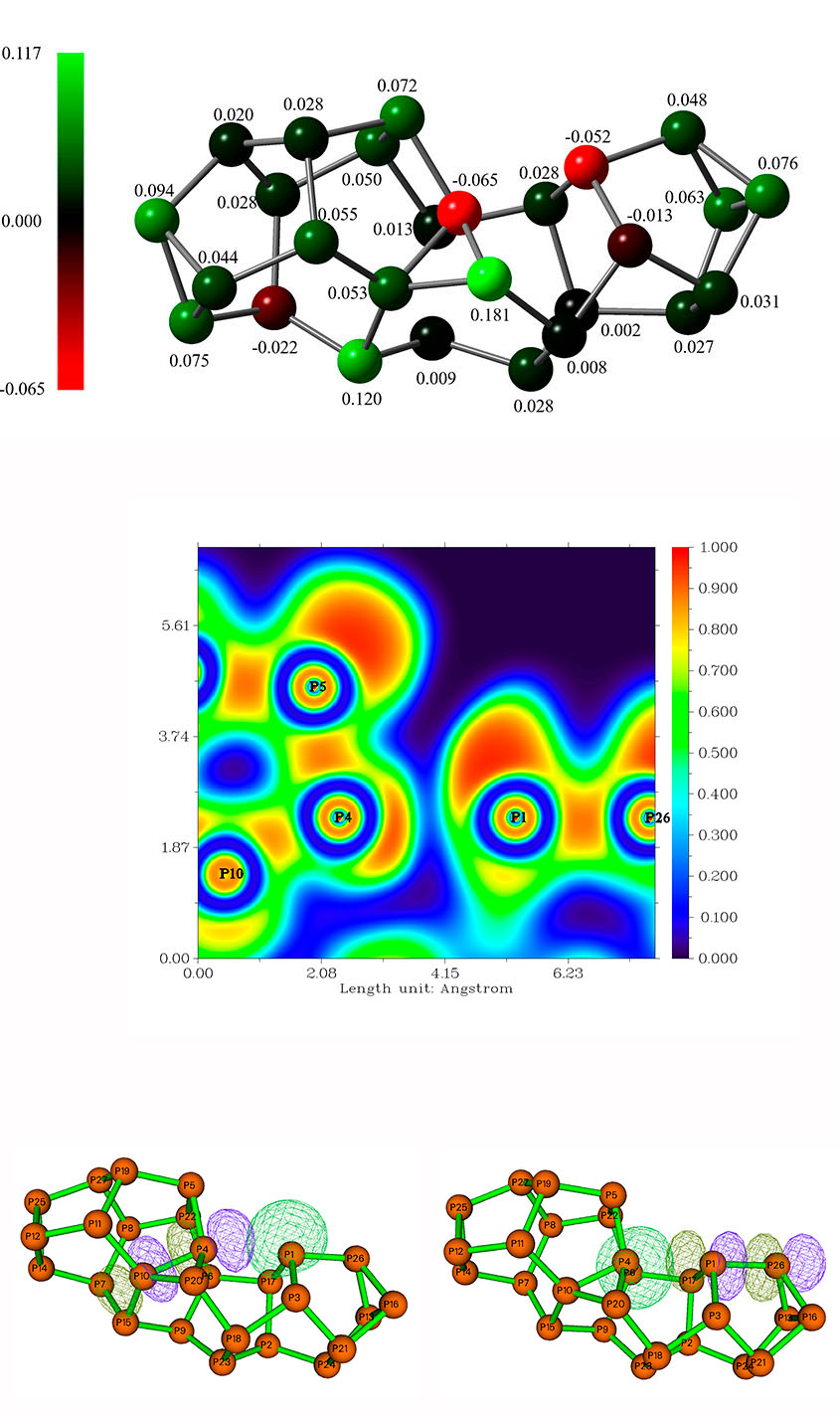


**Fig. S4** Critical points in AIM topology analysis of a)$P_{27}^{+}-I$, b)$P_{29}^{+}-I$ and c) $P_{31}^{+}-I$.


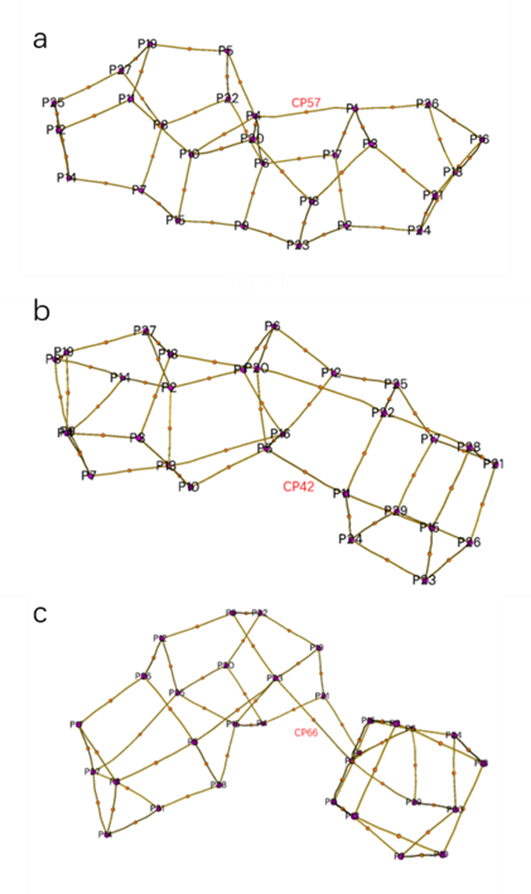


**Table S1** The relative energies of the top three isomers of $P_{2n+1}^{+}$ (n = 12-15) obtained on different methods

| *Structures* | *Relative energies (in kJ/mol) to their corresponding global minima* | |
| --- | --- | --- |
|  | *Method A^a^* | *Method B^b^* |
| $P_{25}^{+}-I$ | *0* | *0* |
| $P_{25}^{+}-\mathrm{II}$ | *80.5* | *81.8* |
| $P_{25}^{+}-\mathrm{III}$ | *93.9* | *112.7* |
| $P_{27}^{+}-I$ | *0* | *0* |
| $P_{27}^{+}-\mathrm{II}$ | *3.5* | *28.6* |
| $P_{27}^{+}-\mathrm{III}$ | *6.6* | *84.8* |
| $P_{29}^{+}-I$ | *0* | *0* |
| $P_{29}^{+}-\mathrm{II}$ | *52.5* | *86.0* |
| $P_{29}^{+}-\mathrm{III}$ | *59.3* | *92.8* |
| $P_{31}^{+}-I$ | *0* | *0* |
| $P_{31}^{+}-\mathrm{II}$ | *26.7* | *24.7* |
| $P_{31}^{+}-\mathrm{III}$ | *27.3* | *26.0* |

^a^ZPE-corrected energies calculated with the method of B3LYP/6-311+G(d)// B3LYP/6-311+G(d).

^b^ZPE-corrected energies calculated with the method of MP2/6-311+G(d)// B3LYP/6-311+G(d).

**Table S2:** The Quantum Theory of Atoms in Molecules (QTAIM) was applied to analyze the pnicogen interaction in $P_{27}^{+}-I , P_{29}^{+}-I and P_{31}^{+}-I$. ***^a^***

| Isomer | Bond | Type***^b^*** | *ρ_e_* ***^c^*** | ▽^2^*ρ_e_****^d^*** | ELF***^e^*** | Sign(*λ_2_*)**ρ****^f^*** | H(r)***^g^*** |
| --- | --- | --- | --- | --- | --- | --- | --- |
| $P_{27}^{+}-I$ | P1…P4 | (3, -1) | 0.0258 | 0.0415 | 0.2245 | -0.0258 | -0.0016 |
| $P_{29}^{+}-I$ | P5…P11 | (3, -1) | 0.0280 | 0.0419 | 0.2545 | -0.0280 | -0.0022 |
| $P_{31}^{+}-I$ | P2…P23 | (3, -1) | 0.137 | 0.0282 | 0.0958 | -0.0137 | 0.0001 |

***^a^***The AIM topology analysis is performed using Multiwfn program^[1]^.

***^b^***The type of critical points (CPs). CPs can be classified into four types according to how many eigenvalues of Hessian matrix of real space function are negative. And the (3,-1) indicates that the two eigenvalues of Hessian matrix of function are negative, namely the second-order saddle point. (3,-1) generally appears between attractive atom pairs and hence commonly called as bond critical point (BCP).

***^c^****ρ_e_* **:** The electron density at CP. The value of *ρ_e_* is related to bonding strength.

***^d^***▽^2^*ρ_e_*: Laplacian of *ρ_e_*.

***^e^***ELF: Electron Localization Function

***^f^****λ_2_* is the second largest eigenvalue of Hessian matrix. The value of sign(*λ_2_*)**ρ* < 0 indicates that there is an attractive weak interaction at the BCP. The value of sign(*λ_2_*)**ρ* gets smaller, the interaction gets stronger ^[2]^. The negative values of sign(*λ_2_*)**ρ* in the table indicate that the pnicogen bond does exist in these structures.

***^g^*** H(r): Energy density.

References

[1] Tian Lu, Feiwu Chen. Multiwfn: A multifunctional wavefunction analyzer. J. Comput. Chem. 2012. 33, 580-592

[2] Johnson ER, Keinan S, Mori-Sánchez P, Contreras-García J, Cohen AJ, Yang WT. Revealing Noncovalent Interactions. J. Am. Chem. Soc. 2010, 132, 6498-6506

**The Cartesian coordinates of the most stable isomers of** $\mathbf{P}_{\boldsymbol{2}\boldsymbol{n+1}}^{\boldsymbol{+}}$ **(n = 1 – 15) (in Angstroms).**

$$P_{3}^{+}$$

P 0.00000000 1.22132100 0.00000000

P 1.05769500 -0.61066000 0.00000000

P -1.05769500 -0.61066000 0.00000000

$$P_{5}^{+}$$

P -0.76419900 1.36090100 -0.34969800

P 1.36093500 0.76423200 -0.34949500

P -1.36086800 -0.76423300 -0.34975500

P 0.76426600 -1.36090100 -0.34955200

P -0.00013500 0.00000000 1.39850100

$$P_{7}^{+}$$

P 1.14274400 1.53398400 -0.00113100

P -1.14274600 1.53398400 -0.00113000

P 1.64664800 -0.35166900 1.08160600

P -1.64664700 -0.35167100 1.08160600

P 0.00000000 -1.65798700 -0.00053500

P -1.64798400 -0.35332100 -1.08020800

P 1.64798400 -0.35332000 -1.08020800

$$P_{9}^{+}$$

P 0.00000000 0.00000000 0.00000000

P 0.00000000 1.55133500 1.59838400

P 0.00000000 -1.55133500 1.59838400

P 1.10435500 0.00000000 2.84999500

P -1.10435500 0.00000000 2.84999500

P 1.55133500 0.00000000 -1.59838400

P -1.55133500 0.00000000 -1.59838400

P 0.00000000 1.10435500 -2.84999500

P 0.00000000 -1.10435500 -2.84999500

$$P_{11}^{+}$$

P 2.76815900 -0.00004300 1.31108800

P 0.48257000 -1.79187500 -0.09317400

P 0.48253900 1.79191400 -0.09327200

P -1.08641600 1.15434100 -1.55343000

P -2.67355000 -1.16939100 0.12500700

P 0.36170300 0.00005100 1.14995100

P 2.60237900 -1.13536600 -0.64192200

P -1.77984700 0.00001800 1.86600500

P -1.08636400 -1.15442300 -1.55340300

P -2.67360100 1.16937200 0.12499900

P 2.60242700 1.13540200 -0.64184900

$$P_{13}^{+}$$

P 0.00000000 -0.33033600 -1.78883800

P -3.17447000 0.39515300 1.15585000

P 3.17446400 0.39516000 1.15585100

P -3.17446700 0.39517200 -1.15585400

P 0.00000000 0.97223000 0.00000100

P 0.00000300 -0.33033200 1.78883400

P -1.81146200 -1.46262900 -1.16405100

P -1.81145100 -1.46264300 1.16404300

P 1.81146100 -1.46263200 -1.16404800

P 1.81145700 -1.46264300 1.16404600

P -2.00324600 1.97916500 0.00001500

P 3.17446700 0.39516900 -1.15585400

P 2.00324500 1.97916700 0.00000400

$$P_{15}^{+}$$

P 1.46833100 -2.01386600 -0.31359700

P -3.56495800 0.05701200 -1.46731400

P -2.29085000 1.77837700 -0.63096700

P -0.47214100 -0.89479200 -0.28243400

P -3.77640200 -0.82018300 0.65638500

P -0.73877100 -0.37600500 1.87153800

P 1.14319900 2.03897100 -0.04342800

P -2.41969000 -1.82125300 -0.89003100

P 3.02385500 1.30346900 -1.07029000

P -2.51540700 0.91254900 1.50644200

P 4.05939200 -0.17093300 0.24176000

P 2.91962500 -0.84960100 -1.68309500

P 1.14134200 0.84817100 1.94270400

P -0.34328200 1.01880700 -1.38987500

P 2.36575800 -1.01072400 1.55220100

$$P_{17}^{+}$$

P -0.92914500 0.35122500 1.67494800

P -0.91283900 0.68738700 -0.52639000

P -3.28190200 -1.54895800 -0.33744900

P -4.27166800 0.35642000 -1.17727800

P 0.40498700 -1.45426900 1.71380900

P 3.35885400 -1.40139900 -0.00029400

P 1.80333600 -1.05884200 -1.59340100

P -2.69554200 1.99038000 -0.93303900

P -3.97990100 1.41558100 0.85531400

P 4.10910000 2.01450800 -0.08589300

P 4.88552500 0.17838500 -0.58619400

P 0.29429100 -2.31273200 -0.48765300

P -2.98630300 -0.48031000 1.69621900

P 2.40849500 -0.34617500 1.73652700

P 1.15346800 1.18832800 -1.24961500

P -1.40194200 -1.24859100 -1.48744700

P 2.04118800 1.66906200 0.78783600

$$P_{19}^{+}$$

P 5.85566300 -0.00009300 0.42881800

P 4.32817600 -1.64904400 0.46474800

P 4.25449200 -0.00035900 2.00440400

P 0.64474100 -0.00021600 1.19518800

P -0.42552500 1.18780800 -1.95056300

P 2.95164300 1.14246500 -1.23981800

P -1.98879800 1.77935600 -0.44734000

P 2.95164400 -1.14201600 -1.24022900

P -1.59412900 -0.00015900 0.82820500

P 1.02106700 1.71635300 -0.29996900

P 1.02106000 -1.71623300 -0.30060100

P -4.02307000 1.15507900 -1.04361800

P -4.02307300 -1.15468400 -1.04404200

P -4.81249600 -1.15780300 1.12328100

P 4.32819800 1.64886900 0.46532700

P -4.81250100 1.15738000 1.12370700

P -1.98880100 -1.77919500 -0.44801200

P -0.42552200 -1.18707500 -1.95100600

P -3.26276900 -0.00043200 2.33152100

$$P_{21}^{+}$$

P 2.35156300 1.60173000 1.32582600

P 4.28338000 -1.49876500 0.93240100

P 2.63430500 0.07015900 -1.95229300

P 1.59876400 -1.79263100 -1.18950500

P -2.29103200 -0.73569100 2.43305100

P -0.65774500 -0.00853800 1.07875300

P -1.02778800 2.17705700 0.76440300

P 2.07938300 -2.03925600 0.99659400

P -3.65976300 -1.42453500 -1.14242300

P -0.95939800 0.63352200 -2.37052900

P -3.14750700 0.74582100 -1.78369100

P -0.52059700 -1.11895200 -1.02030800

P 1.29503500 -0.24090300 2.13307500

P -3.16994300 2.00924700 0.13187000

P 4.44886400 0.76525100 1.16606100

P 2.10321000 1.97002300 -0.88495600

P -4.03391500 -1.36989900 1.10354400

P -1.86026000 -2.70244000 -1.38001900

P -3.96236000 0.72218000 1.86281100

P -0.13501800 2.44140100 -1.28701200

P 4.63082300 -0.20478200 -0.91765200

$$P_{23}^{+}$$

P 2.26626300 -0.76750500 -0.29206200

P 1.81325100 2.34269700 -1.07064800

P 0.06894400 -0.88996400 -0.74130400

P -3.40695600 1.61766800 -0.52595300

P -5.38400900 -2.16529900 -0.01875900

P -3.43713100 -0.21921500 -1.78701900

P 5.51716300 -1.49451700 -1.16181600

P 1.71864100 2.16260000 1.28005000

P -0.50836700 -0.60536100 1.43056400

P -0.49021200 1.62879900 1.68411200

P -5.85263000 -0.11611600 0.82513700

P 2.62053800 0.14984900 1.71426000

P -2.73208500 -1.15817900 1.58932600

P -0.05504600 1.26713900 -1.72280900

P 3.07272200 0.64039600 -1.80137300

P -3.87605000 0.78302500 1.48952100

P 3.64922100 -2.53739800 -0.36973200

P -1.26497000 2.42935200 -0.27919800

P 5.22398100 -1.82453400 1.10772800

P 4.79652500 0.42005700 1.37695000

P -3.14109100 -2.03022900 -0.39944600

P 5.09466500 0.75385600 -0.89233600

P -5.69336700 -0.38711900 -1.43519300

$$P_{25}^{+}$$

P -3.11775600 -1.16908700 -1.82004200

P -1.79934300 -2.82452800 -0.93249300

P 0.24936500 -0.52880100 0.14969000

P 3.57703700 0.02077800 1.38232800

P 1.43655000 3.15611700 -0.65196900

P 1.19044000 2.58776600 1.53652600

P -1.15326000 -2.04204000 1.05147800

P 1.41745900 0.38186000 1.90330100

P -0.36654600 2.32991100 -1.80427000

P -1.53351700 4.52637300 0.75668100

P -2.97647100 -0.83844000 1.65861500

P -1.53635600 4.25686800 -1.49004000

P -4.58962600 -2.36669100 1.20562700

P 3.46698900 -2.20009500 1.60264000

P 5.19431100 -1.25853800 -1.45796000

P 0.30984800 5.14498900 -0.41692700

P 2.95612400 -1.40686800 -1.89645100

P 2.69439600 -3.07415300 -0.30127300

P -3.65038800 -3.91027900 -0.16606100

P 4.93727700 -2.97170700 0.15528600

P 3.79675400 0.33536200 -0.81550100

P -1.03091100 2.54181400 1.70923900

P -4.98184300 -2.25129900 -1.03969700

P -1.20308400 1.18501100 -0.10083900

P -3.28744700 0.37567900 -0.21789000

$$P_{27}^{+}$$

P 2.57852300 1.56730100 0.37620600

P 2.91721400 -1.29178200 -1.81231600

P 3.09995800 -0.02409900 1.96230500

P -0.17386600 0.62228200 0.95920400

P -1.31982500 2.53771900 0.79767800

P 0.11500300 0.38166800 -2.30545100

P -3.33087000 -1.27208700 -1.78170900

P -3.18706800 1.00845700 -2.08385600

P -0.09112900 -1.86772100 -1.68373900

P -1.66118700 -0.91080600 1.27470500

P -3.44221600 0.00344000 2.29329400

P -5.34338000 -0.92259400 1.48361700

P 5.84911800 0.75667400 -1.26979800

P -5.28655100 -1.57663200 -0.71390500

P -1.87570200 -2.35425500 -0.46310000

P 6.14945400 0.99878400 1.01990000

P 2.25587300 0.85487400 -1.76387700

P 1.78154300 -1.84825200 1.83946000

P -3.44403100 2.09038800 1.40819100

P 0.00773800 -0.72982500 2.78415300

P 5.01159200 -0.95577400 1.35650700

P -1.26556000 2.03563500 -1.55536900

P 1.63029100 -2.49700100 -0.39720300

P 4.98682300 -1.33070600 -0.89455100

P -6.13147300 0.45114500 -0.17340100

P 4.67783100 2.28009600 -0.07749700

P -4.50810400 1.99307200 -0.57944800

$$P_{29}^{+}$$

P -1.51991700 2.52335800 -0.65423600

P -3.19027300 1.04862500 -0.52955700

P -2.49903700 -2.35993100 1.39945200

P -4.67230200 -2.31798800 0.64444700

P 0.31081300 -0.55932200 0.05519900

P -0.57261900 2.52194700 1.47189100

P -4.45323300 -1.92599700 -1.60688600

P -6.64594700 0.64603400 -0.18816500

P -5.98522400 -0.31810700 -2.12468300

P -1.30645400 -2.03983800 -0.54363500

P 2.86615300 -1.81296500 0.80507900

P 1.17807200 2.71223400 0.01904100

P -2.59020200 -0.72025300 -1.79405100

P -5.10940400 1.71236600 -1.51450800

P 5.16702600 -2.27816000 1.09779600

P 0.20046500 1.18787500 -1.37428800

P 4.35901000 2.07874000 -0.89675100

P -2.36896000 -0.42095300 2.62140600

P -5.51680900 -0.36937200 1.51471300

P -0.29160500 0.35970500 2.06794700

P 6.19813200 1.07812900 -0.01500800

P 3.21907100 0.09105900 1.98236800

P 5.27837500 -2.31056800 -1.17603200

P 3.09741600 -1.57641400 -1.44128900

P 3.11335000 2.07338700 0.94542300

P 6.09484500 -0.28747600 -1.87092800

P -3.76437000 1.00428900 1.63714100

P 5.49297700 -0.11974900 1.81325500

P 3.91065100 0.37934500 -2.34514200

$$P_{31}^{+}$$

P 0.93450500 3.38733100 -2.02851200

P 1.58538500 1.43860800 -1.08802400

P 4.27089600 -1.88944700 2.51713600

P -2.39389700 2.83164000 -1.58355500

P -1.37734500 -2.19049600 0.53152900

P 2.67899600 3.30822300 -0.59143100

P 4.41776800 -1.30970700 -1.67189800

P -2.48772200 -4.14516400 -0.00438600

P -2.37970600 1.30968600 2.84631400

P 5.86026400 -2.38609200 -0.31058500

P 2.63323900 -1.72074100 -0.32412300

P -4.15482700 0.16091300 2.08788200

P 3.98174600 -3.32021500 0.69444500

P -3.33972800 -3.90974800 -2.18334600

P -2.11617400 0.81604400 -0.56799400

P 2.91090200 -0.32307900 1.47521100

P -4.48715400 -3.25513500 0.71509900

P 6.13328800 -0.92287900 1.49858400

P -0.57631100 3.28751100 1.37929800

P -3.97080100 3.09767000 0.06986800

P -0.78632100 4.08897900 -0.70110700

P -2.76999100 3.42409500 2.05437800

P -0.75681400 1.01483100 1.24512200

P 4.80194700 0.84352500 1.00057200

P -4.28123700 0.83893400 -0.04933500

P -3.18898100 -1.87644800 1.92258600

P -4.99928800 -2.48931100 -1.40231000

P -1.42311600 -1.11416500 -1.48003100

P 3.46054800 1.96514300 -2.24559000

P 5.09102400 0.76859500 -1.21602900

P -3.27109300 -1.72910000 -2.58976600
